# Excitation-Dependent Photoluminescence of BaZrO_3_:Eu^3+^ Crystals

**DOI:** 10.3390/nano12173028

**Published:** 2022-08-31

**Authors:** Santosh K. Gupta, Hisham Abdou, Carlo U. Segre, Yuanbing Mao

**Affiliations:** 1 Radiochemistry Division, Bhabha Atomic Research Centre, Trombay, Mumbai 400085, India; 2 Homi Bhabha National Institute, Anushakti Nagar, Mumbai 400094, India; 3 Department of Chemistry, University of Texas Rio Grande Valley, 1201 West University Drive, Edinburg, TX 78539, USA; 4 Center for Synchrotron Radiation Research and Instrumentation and Department of Physics, Illinois Institute of Technology, Chicago, IL 60616, USA; 5 Department of Chemistry, Illinois Institute of Technology, 3105 South Dearborn Street, Chicago, IL 60616, USA

**Keywords:** BaZrO_3_, europium, luminescence, EXAFS, defect

## Abstract

The elucidation of local structure, excitation-dependent spectroscopy, and defect engineering in lanthanide ion-doped phosphors was a focal point of research. In this work, we have studied Eu^3+^-doped BaZrO_3_ (BZOE) submicron crystals that were synthesized by a molten salt method. The BZOE crystals show orange–red emission tunability under the host and dopant excitations at 279 nm and 395 nm, respectively, and the difference is determined in terms of the asymmetry ratio, Stark splitting, and intensity of the uncommon ^5^D_0_ → ^7^F_0_ transition. These distinct spectral features remain unaltered under different excitations for the BZOE crystals with Eu^3+^ concentrations of 0–10.0%. The 2.0% Eu^3+^-doped BZOE crystals display the best optical performance in terms of excitation/emission intensity, lifetime, and quantum yield. The X-ray absorption near the edge structure spectral data suggest europium, barium, and zirconium ions to be stabilized in +3, +2, and +4 oxidation states, respectively. The extended X-ray absorption fine structure spectral analysis confirms that, below 2.0% doping, the Eu^3+^ ions occupy the six-coordinated Zr^4+^ sites. This work gives complete information about the BZOE phosphor in terms of the dopant oxidation state, the local structure, the excitation-dependent photoluminescence (PL), the concentration-dependent PL, and the origin of PL. Such a complete photophysical analysis opens up a new pathway in perovskite research in the area of phosphors and scintillators with tunable properties.

## 1. Introduction

The trivalent europium ion Eu^3+^ is considered to be one of the most sensitive lanthanide ions that displays environment- and symmetry-sensitive emissions owing to its pure magnetic dipole transition (MDT, ∆J = ±1), hypersensitive electric dipole transition (HEDT, ∆J = ±2), and neither magnetic nor electric ^5^D_0_ → ^7^F_0_ (∆J = 0) transition [1,2,3,4]. When Eu^3+^ is localized at a highly symmetric site with a center of inversion (*C*_i_), its MDT predominates over its EDT. If Eu^3+^ is situated at a highly asymmetric site, its emission is the other way around [5]. In addition, the Eu^3+^ ion is one of the most fascinating dopant ions for quality red phosphors with a high quantum yield (QY), a decent thermal stability, and a long luminescence lifetime [6,7].

Perovskites with a generic formula ABO_3_ are in high demand as luminescence hosts due to their structural flexibility, wide band gap, ease of doping, and ability to accommodate lanthanide ions at both A and B sites [8,9,10]. Among them, BaZrO_3_ (BZO) is a unique material due to its wide tunable band gap (5.6 eV) [11], high refractive index [12], high proton conductivity, and high chemical and mechanical stability [10,13,14,15]. It has various applications in the areas of luminescence [16], catalysis [16,17], proton-conducting solid oxide fuel cells [18,19], and many others. Eu^3+^ ion-doped ABO_3_ perovskites have attracted a lot of attention due to their high thermal and chemical stability, low environmental toxicity, and various applications in photocatalysis, white light generation [20], light emitting diodes (LEDs) [21], and bioimaging [22].

One can probe the local sites of Eu^3+^ ions in ABO_3_ perovskites based on the ratio, spectral splitting, and appearance of ^5^D_0_ → ^7^F_J_ (J = 0–4) emissions.^1^ This kind of study is crucial to make materials with optimum light emitting properties. For example, Kunti et al. recently observed MDT and HEDT emissions with *I*_MDT_ >>> *I*_HEDT_ along with the host emission under the 275 nm excitation from their BaZrO_3_:Eu samples synthesized by the solid state route [23]. There was a systematic host-to-dopant energy transfer with an increasing Eu^3+^ doping concentration. Based on the analysis of extended X-ray absorption fine structure (EXAFS) spectroscopic data, they concluded that Eu^3+^ ions were localized at Zr^4+^ sites [23]. Gupta, one of the co-authors of the current manuscript, and his coworkers observed spectral profiles with *I*_HEDT_ >>> *I*_MDT_ under various excitations from gel combustion-synthesized BZO:Eu samples [12], which was exactly opposite to what was observed by Kunti et al. [23]. Gupta et al. also proposed that a large fraction of Eu^3+^ ions occupied Zr^4+^ sites based on population analysis of lifetime spectra [12]. Kanie et al. synthesized BZO samples with different sizes and shapes and investigated their effects on luminescence [24].

There were also reports on Eu^3+^-doped perovskites of SrZrO_3_, SrSnO_3_, BaTiO_3_, BaSnO_3_, and BaZr_x_Ti_1−x_O_3_. For example, Basu et al. proposed that Eu^3+^ ions resided at Sr^2+^ sites at low dopant concentrations and were distributed at both Sr^2+^ and Sn^4+^ sites at high doping levels in their polyol-synthesized SrSnO_3_ nanoparticles based on EXAFS measurements [25]. The same group further proposed that Eu^3+^ ions occupied the centrosymmetric Sr^2+^ sites up to 1.5% Eu^3+^ doping and, beyond that, the synthesized SrSnO_3_ nanoparticles formed a separate europium oxide phase based on time resolved emission spectroscopy (TRES) and electron paramagnetic resonance (EPR) studies. Similarly, based on EXAFS studies, Rabufetti et al. found that Eu^3+^ ions resided at Ba^2+^ sites at low dopant concentrations (up to 4% Eu^3+^ doping) but were distributed at both Ba^2+^ and Ti^4+^ sites at high doping levels in their vapor-diffusion sol-gel-synthesized BaTiO_3_ nanocrystals [26]. Canu et al. have tuned the photoluminescence properties of Eu^3+^-doped BaZr_x_Ti_1-x_O_3_ perovskite by applying an electric field [27].

There are also reports that studied the effect of changing the A cation of AZrO_3_:Eu on the luminescence emission intensities [28]. Katyayan et al. studied the impact of co-doping Tb^3+^ with Eu^3+^ on the optical and spectroscopic characteristics of BZO perovskite [29]. Another study investigated the effect of particle size and morphology on the fluorescence behaviors of these metal oxides [24].

Color tunability is achieved from samples with the same dopants and hosts by simply varying the excitation wavelength. For example, Gupta et al. showed different emission characteristics of SrZrO_3_:Eu^3+^ nanoparticles in terms of the asymmetry ratio (*A*_21_) under the excitations with host absorption, charge transfer, and the *f-f* band of Eu^3+^ [30]. Guo et al. synthesized a Bi^3+^ and Eu^3+^ ion co-doped Ba_9_Lu_2_Si_6_O_24_ single-phased phosphor via a conventional high-temperature solid-state reaction [31]. They demonstrated that the relative emission intensity of Bi^3+^ luminescent centers tightly depends on the incident excitation wavelength due to the complex energy transfer processes among these Bi^3+^ centers.

Furthermore, even though the induced electric dipole (ED) ^5^D_0_ → ^7^F_0_ transition is strictly forbidden by the ΔJ selection rule of the Judd–Ofelt theory, there are reported occurrences of it as a well-known example of the breakdown of the selection rules of the Judd–Ofelt theory [1]. For example, Guzmán-Olguín et al. showed an unusual great intensity of the ^5^D_0_ → ^7^F_0_ transition centered at 580 nm when they excited their Eu^3+^-doped BaHfO_3_ perovskite ceramic under UV radiation with the wavelength associated with the charge transfer band (272 nm), while this transition was very weak when the sample was excited at 396 or 466 nm wavelengths [32]. One of the co-authors of this manuscript, Gupta, with his co-workers, reported the presence of two Stark components in the ^5^D_0_ → ^7^F_0_ transition from their Nd_2_Zr_2_O_7_:Eu phosphor when excited at 256 nm [33].

It is clear that there is no systematic investigation of the luminescence of BZO:Eu nor studies on its ^5^D_0_ → ^7^F_0_ transition under host and Eu^3+^ excitations. In this work, we have first synthesized BZO:Eu submicron crystals using an environmentally friendly molten salt synthesis (MSS) method based on the report by Zhou et al. using barium oxalate and zirconium oxide as precursors and a KOH/NaOH salt mixture as the reaction medium [34]. We studied tuning the red to orange emission ratio from the BZO:Eu crystals by modulating the excitation wavelength and deciphered the local site occupancy of Eu^3+^ ions in BZO with Eu, Ba, and Zr-edge EXAFS analysis. More importantly, other than the weak ^5^D_0_ → ^7^F_3_ at 653 nm, we observed a strong ^5^D_0_ → ^7^F_0_ transition, which is known to be strictly forbidden by both EDT and MDT of Eu^3+^ ions, as based on the Judd–Ofelt theory. This observation suggests the deviation of luminescence properties of the Eu^3+^ dopant in the BZO host from the Judd–Ofelt theory. In other words, it indicates that Eu^3+^ ions are localized in highly asymmetric environments, e.g., *C*_n_, *C*_nv_, and *C*_s_ point group symmetry, so that the selection rules are relaxed to some extent by the mixing of a low-energy charge transfer state with the 4f^6^ configuration [1]. Moreover, designing functional materials that display excitation wavelength-dependent color tunability and understanding structure–property correlation is invaluable to materials scientists.

## 2. Experimental

The synthesis and instrumentation characterization of the BZO and BZOE submicron crystals are described in detail in the electronic supplementary information as S1. Briefly, six Ba_1-x_ZrO_3_:x%Eu^3+^ (x = 0, 0.5, 1.0, 2.0, 5.0, 10.0) samples were synthesized using the MSS method following a procedure published previously with one of the co-authors of this manuscript. Based on the Eu^3+^ doping levels, the synthesized Ba_1-x_ZrO_3_:x%Eu^3+^ samples with x = 0, 0.5, 1.0, 2.0, 5.0, 10.0 are designated as BZO, BZOE-0.5, BZOE-1, BZOE-2, BZOE-5, and BZOE-10, respectively.

## 3. Results and Discussion

### 3.1. XRD Patterns

**Figure 1:**
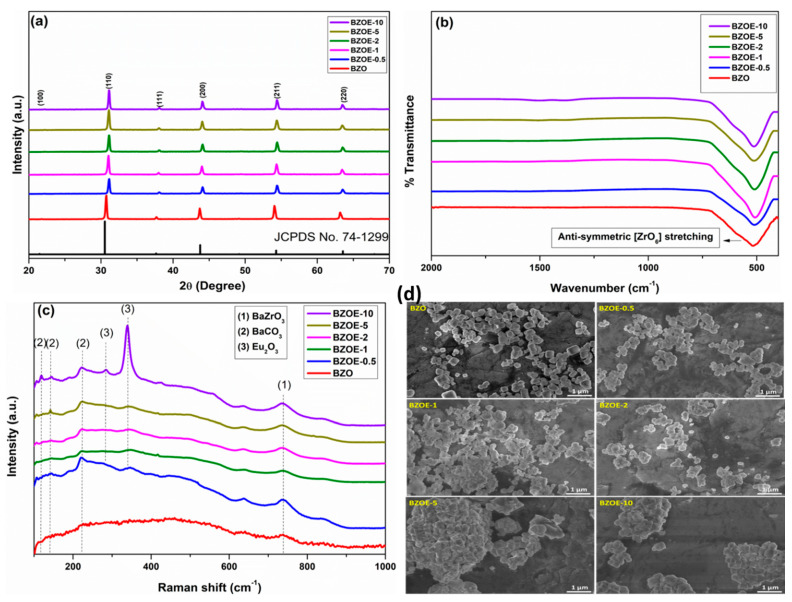
(**a**) XRD patterns, (**b**) FTIR spectra, (**c**) Raman spectra, and (**d**) SEM images of the BZO and BZOE samples.

The XRD patterns of the BZO and BZO:Eu samples (Figure 1a) demonstrated that the diffraction peaks of all samples match with the cubic perovskite phase (*Pm*-3*m*) of BZO (JCPDS No. 74-1299) and no impurity peaks were observed. The substitution of Eu^3+^ for constituent ions is evidently aliovalent and may generate oxygen vacancies when resided at a Zr^4+^ site. In case if some fraction resides at a Ba^2+^ site, the charge compensation may invoke the creation of barium vacancies. As seen in Table 1, the cell parameter variation is complex, which means different defect complex generations at different doping levels.

### 3.2. FTIR and Raman Spectroscopy

To further confirm the formation of the perovskite phase and rule out the formation of other phases, FTIR spectra of the samples were collected (Figure 1b). The only observed peak around 570 cm^−1^ can be assigned to the anti-symmetric stretching Zr–O bond of the octahedral ZrO_6_ unit of the BaZrO_3_ lattice [35,36,37].

In the Raman spectra of the BZO and BZOE samples (Figure 1c), the peak around 600–900 cm^−1^ is attributed to the symmetric stretch (*ν*) of the Zr–O bonds in BaZrO_3_ [23]. With an increasing Eu^3+^ doping level, two extra peaks around 283 and 338 cm^−1^ that correspond to symmetric *A*_g_ and degenerated *F*_g_ modes of the stretching vibrations of the *C*_2_-octahedron (Eu_2_-O) started to appear [38]. This means that Eu^3+^ ions stop going into the BZO lattice and precipitate as a separate phase of Eu_2_O_3_ at the doping concentration of 10.0%, which is similar to what Basu et al. observed from their polyol-synthesized SrSnO_3_ nanoparticles based on EXAFS measurements [25]. The peaks between 100 and 230 cm^−1^ can be assigned to BaCO_3_ impurity, which did not show up in the XRD patterns and FTIR spectra due to a low percentage. The carbonate phase probably resulted from the chemisorption of atmospheric CO_2_ on the surface of the BZO crystals upon its exposure to air. It was reported that the existence of such an impurity phase has no effect on the luminescence properties of the BZO perovskite [8].

### 3.3. SEM Images

The SEM images of the BZO and BZOE samples (Figure 1d) demonstrated that the particles were composed of a mixture of spheres and cubes with well-defined edges. In our earlier work, we found that cubic BZO microcrystals predominated when the synthesis was conducted at a higher annealing temperature. There was almost an equal number of spherical and cubical particles from these samples. No difference in the shape of the particles was noticed from these samples with different Eu^3+^ doping concentrations. However, the agglomeration of the particles increased with an increasing Eu^3+^ concentration. Based on the particle size distribution histograms of these samples obtained using the ImageJ software (Figure S1) and the crystallite sizes obtained from the XRD data (Table 1, Figure S2), no clear correlation between the average particle size and the Eu^3+^ doping concentration was established.

### 3.4. X-ray Absorption Spectroscopy

#### 3.4.1. XANES

**Figure 2:**
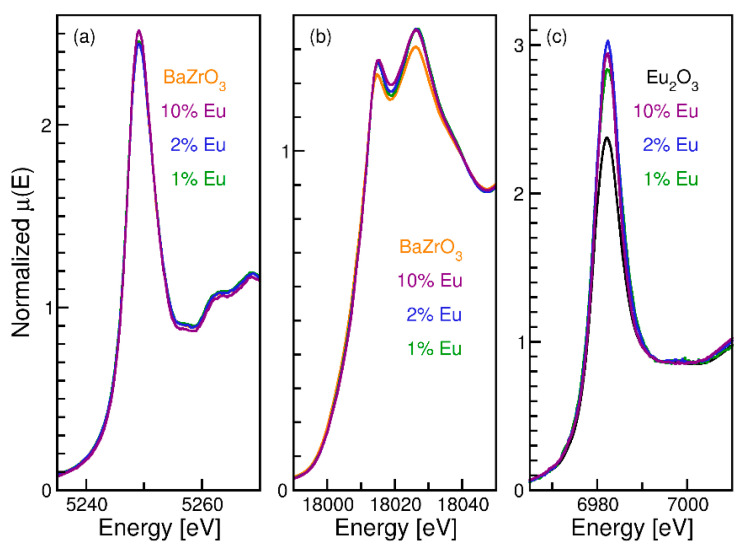
Normalized XANES spectra of the BZOE samples and standards at the (**a**) Ba L_3_-edge, (**b**) Zr K-edge, and (**c**) Eu L_3_-edge.

Figure 2a–c shows the normalized XANES spectra of three BZOE samples along with their standards (either the undoped BZO crystals or commercial Eu_2_O_3_ powder) at the Ba L_3_ (Figure 2a), Zr K (Figure 2b), and Eu L_3_ (Figure 2c) edges, respectively. The normalized XANES spectra at the Ba L_3_-edge shown in Figure 2a are characterized by a sharp white line, which is the main absorption peak due to the transition 2p_3/2_ → 5d. There is no appreciable difference in this edge upon Eu^3+^ doping.

It can be seen from Figure 2b that the Zr absorption edges of the BZOE samples coincide with that of the BZO sample, confirming that the oxidation state of Zr is 4+ in the doped samples. Two peaks, A (18,010 eV) and B (18,021 eV), observed in the BZO and BZOE samples just above the Zr absorption edge, are similar to those obtained by Fassbender et al. [39] and Giannici et al. [40] and are due to the octahedral oxygen coordination of Zr^4+^ in the samples. The overall shape of the Zr XANES spectra remains nearly unchanged upon Eu^3+^ doping, and it is suggested that the octahedral symmetry of the Zr atom does not break with doping. The slight increase in the A and B peaks of the doped samples compared to BZO suggest that the Eu^3+^ solubility is very limited (no more than 1–2%).

The Eu L_3_-edge XANES spectra of the samples (Figure 2c) show that the absorption edges coincide with that of the standard Eu_2_O_3_ sample, suggesting that the Eu dopant remains in the Eu^3+^ oxidation state in the BZOE samples. The increase in the white line at the edge indicates an increase in the empty Eu d-states at the Fermi level in the BZOE samples compared to Eu_2_O_3_, suggesting that the Eu^3+^ is at least partially in a different local environment than in Eu_2_O_3_.

#### 3.4.2. EXAFS

**Figure 3:**
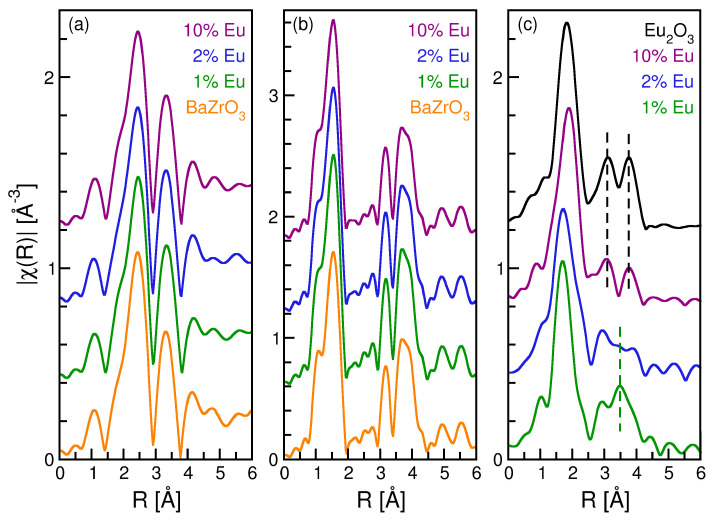
Fourier transformed spectra of the BZOE samples and standards at the (**a**) Ba L_3_ edge, (**b**) Zr K edge, and (**c**) Eu L_3_ edge. Spectra are shifted vertically for clarity.

Figure 3 presents the *k*^2^-weighted Fourier transformed spectra, |*χ*(*R*)|, of the BZO and BZOE samples (and Eu_2_O_3_ standard) at the Ba L_3_ (Figure 3a), Zr K (Figure 3b), and Eu L_3_ (Figure 3c) edges. For BZO in a cubic perovskite structure (ABO_3_) with the space group *Pm*-3*m*, the Zr atoms are coordinated with 6 O atoms in a regular octahedral (6-fold (ZrO_6_)) shape and the Ba atoms are coordinated with 12 O atoms in a cuboctahedral (12-fold (BaO_12_)) shape in the first coordination shells. Theoretical EXAFS spectra have been generated using the above structure for the Ba (Figure S3), Zr (Figure S4), and Eu (Figure S5) edges of the BZO and BZOE samples and fitted to the experimental data.

Specifically, the Ba edge was fitted with a structural model including three paths, Ba–O (12-fold), Ba–Zr (8-fold), and Ba–Ba (6-fold). For the Ba edge fits, the path degeneracies were held constant and the σ^2^ of the Ba–Zr and Ba–Ba paths were constrained to be identical. Windows of 2.0 Å^−1^ < *k* < 8.5 Å^−1^ with d*k* = 2.0 and 1.5 Å < R < 3.7 Å with d*R* = 0.2 were used for the Fourier transformation and fits, respectively. The fit results for the Ba–O paths of each sample are presented in Table 2 and they indicate that the Ba environment is unchanged by Eu doping. The results for the other paths are in Table S1.

The Zr edges were modeled out to 4 Å using three single scattering paths, Zr–O (6-fold), Zr–Ba (8-fold), and Zr–Zr (6-fold), plus the three high amplitude linear multiple scattering paths. The path degeneracies were held constant for all paths and all the multiple scattering paths are constrained to have the same Δ*R* as the Zr–Zr path and a common *σ*^2^ parameter. Windows of 2.0 Å^−1^ < *k* < 13.0 Å^−1^ with d*k* = 2.0 and 1.0 Å < *R* < 4.3 Å with d*R* = 0.2 were used for the Fourier transformation and fits, respectively. Table 2 reports the fit results for the Zr–O single scattering path with the other paths reported in Table S2. For all samples, the Ba–Zr and Zr–Ba paths are distances and disorder parameters and are consistent as are the Ba–Ba and Zr–Zr paths. The quality of the Zr edge fits (Figure S4) are limited by the constraints applied but are consistent across all samples, suggesting only limited Eu^3+^ doping at the Zr site.

The Eu L_3_ EXAFS presented in Figure 3c clearly show that the 10.0% doped sample exhibits two peaks at ~3.1 Å and ~3.7 Å, which are characteristic of Eu_2_O_3_. As the doping level is reduced, these two peaks vanish to be replaced by two small peaks at ~3.0 Å and ~3.5 Å, similar to those observed in the Zr edge EXAFS. The Eu edges were modeled only to the first shell, as the attempts to model the longer paths were unsuccessful. The amplitude reduction factor S_0_^2^ was held constant at the value determined by fitting the Eu_2_O_3_ data. The path length, degeneracy, and disorder of the single Eu–O path being modeled were allowed to vary. Windows of 2.0 Å^−1^ < *k* < 9.0 Å^−1^ with d*k* = 2.0 and 1.0 Å < R < 2.4 Å with d*R* = 0.2 were used for the Fourier transformation and fits, respectively (Figure S5). The resulting fit parameters are reported in Table 2 and it is clear that the Eu–O distance and the path degeneracy increase with the doping level. At 1.0% doping, the Eu–O distance is 2.25 Å, which is longer than the Zr–O distance but significantly shorter than both the Ba–O and the Eu–O distances in Eu_2_O_3_. As the doping level increases, the Eu-O distance increases and then exceeds that found in Eu_2_O_3_. Similarly, the path degeneracy for the 1.0% sample is close to six, as would be expected for doping on the Zr site and increases to a value greater than that in Eu_2_O_3_. These results strongly suggest that Eu^3+^ at low doping levels sits at the Zr site and has a solubility limit between 1.0–2.0%. At higher concentrations, Eu^3+^ ions are found in a Eu_2_O_3_-like local environment. This result is consistent with the change in the white line of the Eu XANES, which increases for 1.0% and 2.0% but decreases for 10%.

### 3.5. PL Spectra

**Figure 4:**
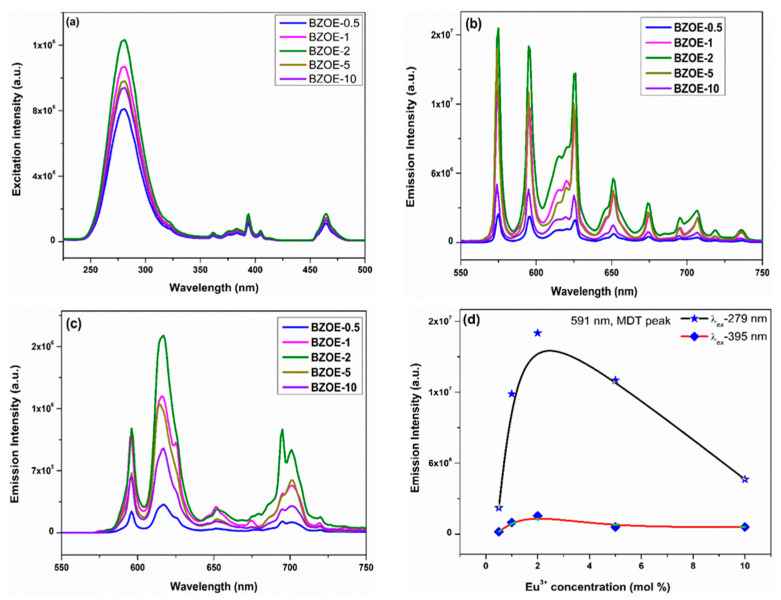
(**a**) Excitation spectra with λ_em_ = 625 nm, emission spectra under (**b**) λ_ex_ = 279 nm and (**c**) λ_ex_ = 395 nm of the BZOE samples. (**d**) Effects of Eu^3+^ doping concentration of the BZOE samples on integrated MDT emission intensity of ^5^D_0_ → ^7^F_1_ transition.

The concentration-dependent excitation spectra (*λ*_em_ = 625 nm, Figure 4a) and emission spectra (*λ*_ex_ = 279 nm and 395 nm, Figure 4b,c) of the BZOE samples demonstrated characteristic PL features of the Eu^3+^ dopant in solid-state hosts [41,42]. In general, there is no change of the excitation and emission spectral profiles, Stark splitting, and relative intensity of excitation and emission peaks under the same excitation wavelength among the BZOE samples with the tested Eu^3+^ doping concentrations. The spectra also clearly show that the 2.0% Eu^3+^-doped sample, BZOE-2, has the highest emission intensity among our samples using the MDT ^5^D_0_→^7^F_1_ as an example (Figure 4d).

The excitation spectra of the BZOE samples with *λ*_em_ = 625 nm corresponding to the ^5^D_0_ → ^7^F_2_ transition of Eu^3+^ ions consisted of two main features (Figure 4a): a broad band extending from 240–320 nm and several fine peaks in the range of 350–500 nm. The broad band peaking around 279 nm is attributed to the allowed charge transfer band (CTB) of electrons from the filled 2p orbital of O^2-^ to the vacant 4d-orbital of the Eu^3+^ ion. The fine peaks around 361, 375, 383, 387, 395, 405, 414, 456, 465, and 472 nm are attributed to the intra f-f transitions of Eu^3+^ ions. The main peaks located at 395 nm and 465 nm are attributed to ^7^F_0_ → ^5^L_6_ and ^7^F_0_ → ^5^D_2_, respectively.

Under the excitations of *λ*_ex_ = 279 and 395 nm, the emission spectra of the BZOE samples displayed the CTB and several fine peaks corresponding to ^5^D_0_ → ^7^F_J_ (J = 0–4) transitions of Eu^3+^ in the spectral range of 550–750 nm (Figure 4b). Interestingly, several significant differences in terms of the appearance of ^5^D_0_ → ^7^F_0_ transition, the asymmetry ratio (*A*_21_), and Stark splitting were observed from the emission spectra of the BZOE samples in the spectral range of 550–750 nm under these two excitation wavelengths.

**Figure 5:**
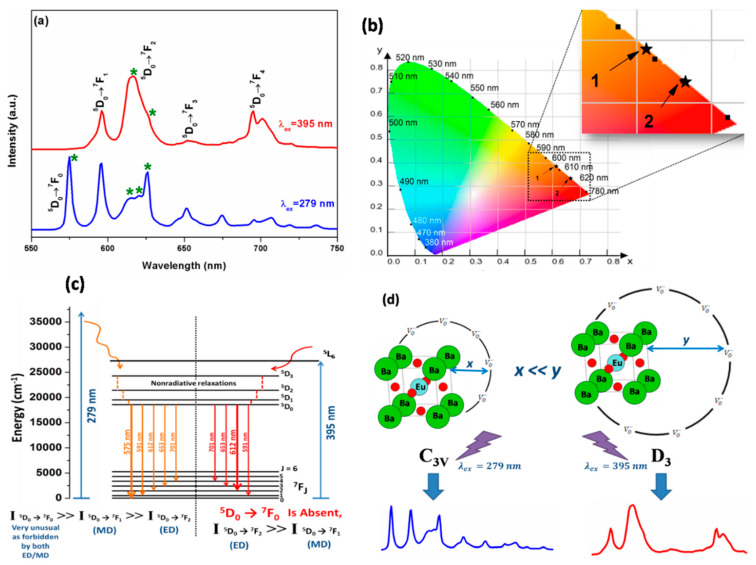
(**a**) Emission spectra of the BZOE-2 sample recorded at 77 K and (**b**) corresponding color coordinate diagram of the BZOE-2 sample under 279 and 395 nm excitations with * indicating the Stark components in (**a**) and arrows 1 and 2 pointing to the coordinates in (**b**), respectively. (**c**) Proposed photophysical processes happening under 279 and 395 nm excitations. (**d**) Schematic showing site selective excitations under 279 nm and 395 nm for the BZOE submicron crystals.

Figure 5a shows a close look of the emission spectra using the BZOE-2 sample as an example. Specifically, under the 395 nm excitation, intense emission bands at 591 nm (^5^D_0_ → ^7^F_1_, MDT), 612 nm (^5^D_0_ → F_2_, HEDT), 701 nm (^5^D_0_ → ^7^F_4_), and 653 nm (^5^D_0_ → ^7^F_3_, weak peak) were observed [43]. There is no signature of ^5^D_0_ → ^7^F_0_ transition. The integral intensity of the HEDT at 612 nm is stronger than that of the MDT at 591 nm. The ^5^D_0_ → ^7^F_3_ transition is known to be allowed by neither MDT nor EDT. It is forbidden in nature according to the Judd–Ofelt (J–O) theory but could gain intensity via J-mixing. The ^5^D_0_ → ^7^F_4_ transition is also considered as an ED transition [1].

Under the CTB excitation at 279 nm, we observed several interesting emission features compared to the emission spectrum recorded under 395 nm excitation (Figure 5a): (a) an unusually intense 575 nm peak corresponding to ^5^D_0_ → ^7^F_0_ transition, which otherwise is forbidden by both ED and MD transitions [44], (b) increased Stark splitting, (c) enhanced intensity of the ^5^D_0_ → ^7^F_3_ peak, and (d) a significant change of the *A*_21_ value. The possible reasons for these observations will be further discussed in the following sections.

### 3.6. PL Lifetime Spectra and QY

**Figure 6:**
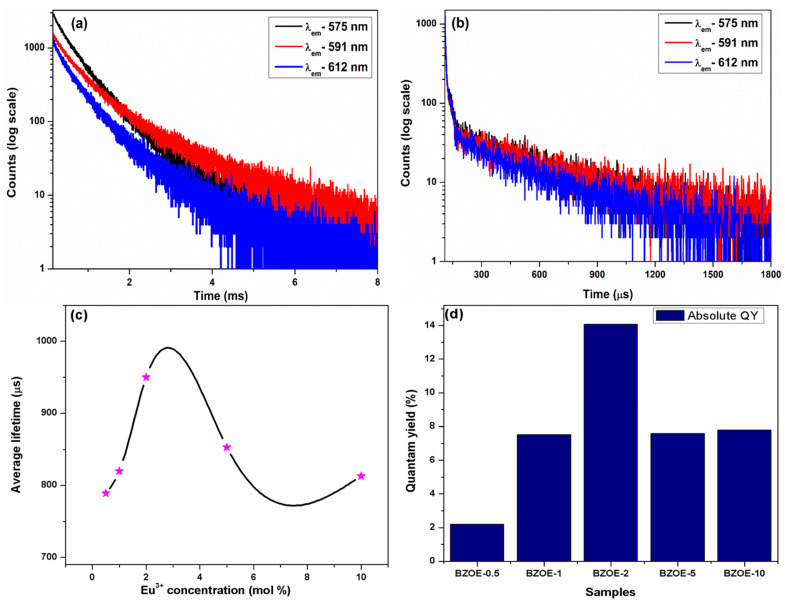
PL decay profiles of the BZOE-2 sample under excitation wavelengths of (**a**) 279 nm and (**b**) 395 nm at three different emission wavelengths of 575, 591, and 612 nm corresponding to ^5^D_0_ → ^7^F_0_, ^5^D_0_ → ^7^F_1_, and ^5^D_0_ → ^7^F_2_ transitions of Eu^3+^ ions, respectively. (**c**) Effects of Eu^3+^ doping concentration of the BZOE samples on (**c**) average lifetime with * indicated the studied Eu^3+^ concentration (mol %) and (**d**) quantum yield (λ_ex_ = 279 nm and λ_em_ = 625 nm).

Figure 6a,b show the results of the luminescence lifetime measurements of the BZOE-2 sample under the excitations at 279 and 395 nm with three different emission wavelengths of 575, 591, and 612 nm corresponding to ^5^D_0_ → ^7^F_0_, ^5^D_0_ → ^7^F_1_, and ^5^D_0_ → ^7^F_2_ transitions, respectively. For the BZOE-2 sample, the luminescence lifetime curves recorded under the 279 nm excitation (Figure 6a) demonstrated a biexponential behavior with two slopes and they can be approximated using the following equation:

*I* = *A*_0_ + *A*_1_ exp(−*t*/*τ*_1_) + *A*_2_ exp(−*t*/*τ*_2_) (1)

where *A*_1_ and *A*_2_ are the derived preexponential factors, and *τ*_1_ and *τ*_2_ are the lifetime values of the fast and slow decay components, respectively. The luminescence lifetime curves recorded under the 395 nm excitation (Figure 6b) could be fitted with monoexponential decay.

The population of Eu^3+^ ions with a particular lifetime is obtained by using the formula:

% of species *n* = (*A*_n_ ∗ *τ*_n_)/(Σ_n = 1,2_*A*_n_ ∗ *τ*_n_)] ∗ 100 (2)

Under *λ*_ex_ = 279 nm, there were two lifetime values for all three emissions: short lifetime *T*_s_ (~360–460 µs, 15%) and long lifetime *T*_l_ (~1.0–1.5 ms, 85%). On the other hand, under *λ*_ex_ = 395 nm, only one short lifetime value was obtained as *T*_s_ (~370–580 µs).

The decay profiles of all other BZOE samples are mentioned in Figure S6. Under λ_ex_ = 279 nm and λ_em_ = 625 nm, the average lifetime values of the BZOE samples with Eu^3+^ doping levels of 0.5, 1.0, 2.0, 5.0, and 10.0% were 789, 820, 950, 853, and 813 µs, respectively. The effect of Eu^3+^ concentration on the average lifetime value of the BZOE samples (Figure 6c) indicated that the average lifetime value increased up to a 2.0% Eu^3+^ doping level. Beyond that doping concentration, there was a reduction due to concentration quenching, which is consistent with the phenomenon observed from the PL excitation and emission spectra shown in Figure 4.

Quantum yield (QY) is an important parameter to evaluate the properties and application potentials of phosphors. We measured and calculated the QY of our BZOE samples using the following equation:

(3) $$\mathrm{QY}=\frac{\int F_{S}}{\int L_{R}+\int L_{S}}$$

where *F*_S_ represents the emission spectrum of a sample, *L*_R_ is the excitation spectrum from an empty integrating sphere (without any sample), and *L*_S_ means the excitation spectrum of a sample. The effect of the Eu^3+^ concentration on the QY value of the BZOE samples (Figure 6d) indicated that the QY value of the BZOE samples increased from 2.2% to 14.0% as the Eu^3+^ doping level increased from 0.5% to 2.0%. After higher dopant concentrations, the QY value reduced to ~7.6–7.9%. This is again attributed to concentration quenching arising from non-radiative energy transfer among Eu^3+^ ions at high doping concentrations.

Concentration quenching is one of the most dominant phenomena that takes place at high dopant concentrations. It is attributed to increasing resonant energy transfer between Eu^3+^ ions at a high dopant concentration, which results in decreasing radiative emissions. To better understand the mechanism of the concentration quenching phenomenon of our BZOE samples, the critical distance (*r*_c_) between Eu^3+^ dopant ions and quenching sites was calculated using the following equation:

(4) $$r_{c}=2{\left(\frac{3V}{4\pi X_{c}N}\right)}^{\frac{1}{3}}$$

where *V*, *X*_c_, and *N* are the volume of the unit cell, the critical concentration of Eu^3+^ and the number of cations per unit cell, respectively. The values of these three variables for our BZOE samples are 73.6575 Å^3^, 0.02, and 8, respectively. Hence, the calculated critical distance *r*_c_ value was 9.58 Å. Since the Eu^3+^–Eu^3+^critical distance is more than 5 Å, multipolar interactions are responsible for the concentration quenching of our BZOE crystals. Therefore, various PL studies indicated that there is a close correlation between the doping concentration with the excitation and emission intensity, luminescence lifetime, and the QY of the BZOE crystals.

### 3.7. J–O Analysis

To explain the observed luminescence performance, J–O parameters were determined to provide empirical relations between the local site symmetry of Eu^3+^ ions in the BZO lattice, the crystal field strength of the BZO host lattice, and the Eu–O bond covalency and polarizability in the BZOE samples.^45^ Based on various mathematical formulations, we have derived the radiative/non-radiative transition rates and the internal quantum efficiency of the BZOE-2 sample [45,46,47]. Various important optical parameters were calculated for the BZOE samples under the excitations at 279 and 395 nm (Table 3). The BZOE-2 sample had a higher internal quantum efficiency (IQE) under the 279 nm excitation compared to 395 nm excitation. Its non-radiative transition (*A*_NR_, 787.4 s^−1^) and radiative transition (*A*_R_, 212.77 s^−1^) values under 279 nm were lower compared to those under 395 nm excitation (*A*_NR_ = 2331 s^−1^ and *A*_R_ = 369 s^−1^). When changing *λ*_ex_ from 279 nm to 395 nm, the increase in the *A*_NR_ value was higher than that of the *A*_R_ value.

For the J–O parameters, *Ω_2_* (the short range parameter) gives information related to the covalent character, local symmetry, and structural distortion in the vicinity of Eu^3+^ ions, whereas *Ω*_4_ intensity parameters (the long range parameter) provides bulk information such as the viscosity and rigidity of the host lattice [48]. Under the 279 nm excitation, the observed trend of the J–O parameters (*Ω*_4_ < *Ω*_2_) suggested that excited Eu^3+^ ions were mostly localized in a highly asymmetric and distorted environment. On the other hand, under the 395 nm excitation, the J–O parameter trend reversed with *Ω*_4_ > *Ω*_2_, which confirmed that a large fraction of excited Eu^3+^ ions occupies relatively less distorted and asymmetric sites. The value of the J–O ratio (*Ω_2_*/*Ω*_4_) of lower than one suggests a high asymmetry of the Eu^3+^ environment where its value higher than one suggests a low asymmetry. The fractional distribution of branching ratios suggests that, under the 279 and 395 nm excitations, photon parts emitted via MDT are 23.5% and 13.6%, whereas those emitted via HEDT are 42.4% and 53.7%, respectively.

### 3.8. Discussion

As marked by numbers one and two on the color coordinated diagram (Figure 5b), the intense peaks around 575 nm and 612 nm impart orange emissions under *λ*_ex_ = 279 nm and red emissions under *λ*_ex_ = 395 nm, respectively. It demonstrated that one can achieve orange–red color tunability by selectively exciting the same material with dopant or host excitations. The different photophysical processes happening under these two excitations are schematically depicted in Figure 5d. The different spectral features of the BZOE samples observed under the 279 and 395 nm excitations suggest that the excited Eu^3+^ ions are relaxed through different channels to the ground states.

Some authors have proposed theoretical models for the observed ^5^D_0_ → ^7^F_0_ transition, including the breakdown of the closure approximation in the Judd–Ofelt theory and third order perturbation theory [1,32,33]. The most obvious explanation assumes that this transition is due to J-mixing or to the mixing of low-lying charge-transfer states into the wave functions of the 4f^6^ configuration. Experimentally, the number of Stark components of the ^5^D_0_ → ^7^F_0_ transition indicates the number of local sites of Eu^3+^ ions in host lattices. It is normally allowed when Eu^3+^ ions are situated at sites lacking inversion symmetry [1,49]. The presence of an unsplitted single band of the ^5^D_0_ → ^7^F_0_ transition under *λ*_ex_ = 279 nm suggests that a large fraction of Eu^3+^ ions are located at the non-inversion symmetric sites in the BZOE submicron crystals. This hypothesis is further supported by the appearance of forbidden ^5^D_0_ → ^7^F_3_ peaks with large Stark splitting [30,44]. On the other hand, under *λ*_ex_ = 395 nm, the observed phenomena, including the absence of ^5^D_0_ → ^7^F_0_ transition, weak ^5^D_0_ → ^7^F_3_ transition, and a low extent of Stark splitting of ^5^D_0_ → ^7^F_1_ and ^5^D_0_ → ^7^F_2_ transitions, suggest that a large fraction of Eu^3+^ ions at doping sites, which are less asymmetric or distorted, are selectively excited.

Based on the selection rules of point group symmetry, the ^5^D_0_ → ^7^F_0_ transition appears when Eu^3+^ dopants are located at sites lacking an inversion center with 10 designated non-cubic point groups, including *C*_6v_, *C*_6_, *C*_3v_, *C*_3_, *C*_4v_, *C*_4_, *C*_2v_, *C*_2_, *C*_s_, and *C*_1_ [50]. The ^5^D_0_ → ^7^F_0_ transition is not allowed in cubic groups with inversion symmetry such as *T*, *T*_d_, and *O* or non-cubic point groups without inversion symmetry such as *D*_2_, *D*_3_, *D*_3h_, *C*_3h_, *D*_3_, *D*_4_, *S*_4_, *D*_2d_, *D*_4d_, and *D*_6_ [49].

The ideal BZO is a perfect cubic perovskite with *O*_h_ point group symmetry (space group: *Pm*-3*m*), which has 12-coordinated Ba^2+^ sites and 6-coordinated Zr^4+^ sites in cuboctahedra and octahedral geometries, respectively [51]. The observed emission spectra are in line with Eu^3+^ ions occupying the Zr^4+^ sites in the BZOE samples even with the following ionic radii values of Ba^2+^ (*r*_ion_ = 161 pm @ CN = 12), Zr^4+^ (*r*_ion_ = 72 pm @ CN = 6), and Eu^3+^ ions (*r*_ion_ = 95 pm @ CN = 6). Substituting Eu^3+^ ions at the Zr^4+^ sites distorts the symmetric ideal perovskite structure of BZO and invokes charge compensation by oxygen vacancies, which reduce the point group symmetry from *O*_h_ to further lower symmetry. This is consistent with our EXAFS analysis (Figure 3), especially at a low Eu^3+^ doping level before the low amount of Eu_2_O_3_ phase forms.

It has been reported that the emission of Eu^3+^ dopant in a cubic structure with the *O*_h_ point group should only have a single unsplitted ^5^D_0_ → ^7^F_1_ transition peak [52]. By considering the most sensitive peaks for ^5^D_0_ → ^7^F_0_ and ^5^D_0_ → ^7^F_2_ transitions, there are 0 and 2 Stark components under *λ*_ex_ = 395 nm and 1 and 3 Stark components under *λ*_ex_ = 279 nm, respectively (Figure 5a). This observation suggested *D*_3_ and *C*_3V_ point group symmetry around Eu^3+^ ions in our BZOE samples [52].

The Kroger–Vink notation for the substitution, wherein trivalent Eu^3+^ ions occupy tetravalent Zr^4+^ sites, is formulated below [53]:

(5) $$2Eu^{˙˙˙}+{Zr}_{Zr}^{˙˙˙˙}↔Eu_{Zr}^{\prime }+V_{O}^{˙˙}$$

Defects such as $V_{O}^{˙˙}$ and $Eu_{Zr}^{\prime }\;$in the BZOE crystals provide additional pathways for non-radiative relaxation. They tend to quench PL by absorbing emitted photon energy from Eu^3+^ ion centers ($Eu_{Ba}^{˙}$) [47]. Hence, although Eu^3+^ ions occupy Zr^4+^ sites ($Eu_{Zr}^{\prime })$, we assume that there are enough oxygen vacancies surrounding them with random distribution. There would be two scenarios: one with enough $Eu_{Zr}^{\prime }$ surrounded by oxygen vacancies in a close vicinity (x), designated as x$Eu_{Zr}^{\prime }$, and another with $Eu_{Zr}^{\prime }$ surrounded by oxygen vacancies at a much farther-off distance (y), designated as y$Eu_{Zr}^{\prime },$ such as y >> x. The point group symmetry of x$Eu_{Zr}^{\prime }$, as discussed above, is *C*_3v,_ and that of y$Eu_{Zr}^{\prime }$ is *D*_3_. As schematically shown in Figure 5d, upon the excitation with the Eu^3+^ *f-f* band at 395 nm, the prevalent excited species is y$Eu_{Zr}^{\prime }$, whereas upon excitation with the host CTB selectively, a large fraction excited species is x$Eu_{Zr}^{\prime }$.

## 4. Conclusions

In this work, BZOE submicron crystals with varied Eu^3+^ doping concentrations were synthesized using the molten salt method. XANES and EXAFS spectroscopies confirm that Eu is stabilized in a +3 oxidation state at Zr^4+^ s at a low doping concentration, while a separate Eu_2_O_3_ phase forms at the highest 10% doping level. Based on the PL measurement, it was established that europium is localized at Zr^4+^ sites in two different environments: one close to zirconium vacancies with *C*_3v_ symmetry and one far off from zirconium vacancies with *D*_3_ symmetry. Interestingly, when excited at the charge transfer band of the BZO host at 279 nm, a large fraction of Eu^3+^ ions at non-symmetric *C*_3v_ sites were excited to give a highly intense ^5^D_0_ → ^7^F_0_ transition, large spectral splitting, and intense MDT peaks compared to HEDT peaks. On the other hand, when excited at a dopant transition wavelength of 395 nm, a relatively large fraction of Eu^3+^ dopants, which are far off from zirconium vacancies with *D*_3_ symmetry, were excited to give no ^5^D_0_ → ^7^F_0_ transition, highly intense HEDT peaks compared to MDT peaks, and fewer Stark components. This excitation wavelength dependence induces emission light tunability of orange light at λ_ex_ = 279 nm and red light at λ_ex_ = 395 nm from the BZOE samples. This observation is further justified by the trend of the J–O parameters, especially with *Ω*_4_ < *Ω*_2_ at λ_ex_ = 279 nm and *Ω*_4_ > *Ω*_2_ at λ_ex_ = 395 nm. This work demonstrates the role of local dopant sites, defects, excitation wavelengths, and doping concentrations on optimizing the optical properties of lanthanide-doped perovskite phosphors for efficient optoelectronics and scintillator applications.

## Figures and Tables

**Table 1:** Lattice constants and crystallite sizes of the BZOE obtained from Rietveld refinement of the XRD data shown in Figure 1a.

%Eu	0.0	0.5	1.0	2.0	5.0	10.0
*a* (Å)	4.1947 (2)	4.1954 (3)	4.1944 (3)	4.1952 (3)	4.1976 (3)	4.1971 (2)
Size (nm)	156 (5)	127 (4)	111 (3)	127 (3)	102 (2)	162 (5)

**Table 2:** Values of the amplitude reduction factor (*S*_0_^2^) or path degeneracy (N), bond length, and disorder factor for the near neighbor paths obtained from EXAFS analysis of the BZO and BZOE samples at the Ba L_3_, Zr K, and Eu L_3_ edges.

Scattering Path	Parameter	BZO	BZOE-1	BZOE-2	BZOE-10
Ba–O N = 12	*S*_0_^2^	0.74 ± 0.16	0.78 ± 0.18	0.80 ± 0.17	0.78 ± 0.17
	*R* (Å)	2.91 ± 0.02	2.91 ± 0.02	2.91 ± 0.02	2.91 ± 0.02
	*σ* ^2^	0.011 ± 0.005	0.013 ± 0.005	0.013 ± 0.005	0.013 ± 0.005
Zr–O N = 6	S_0_^2^	0.90 ± 0.10	1.0 ± 0.1	1.0 ± 0.1	1.1 ± 0.1
	*R* (Å)	2.10 ± 0.01	2.10 ± 0.01	2.10 ± 0.01	2.11 ± 0.01
	*σ* ^2^	0.004 ± 0.001	0.005 ± 0.002	0.005 ± 0.002	0.006 ± 0.002
**Scattering Path**	**Parameter**	**Eu_2_O_3_**	**BZOE-1**	**BZOE-2**	**BZOE-10**
Eu–O *S*_0_^2^ = 0.86	N	7	6.7 ± 1.7	9.8 ± 1.9	7.9 ± 0.7
	*R* (Å)	2.35 ± 0.01	2.27 ± 0.03	2.33 ± 0.02	2.39 ± 0.01
	*σ* ^2^	0.012 ± 0.002	0.012 ± 0.002	0.012 ± 0.002	0.012 ± 0.002

**Table 3:** Calculated J–O parameters and radiative properties of the BZOE-2 sample (*A*_R_ = radiative Rate, *A*_NR_ = nonradiative rate, *Ω_n_* = the Judd−Ofelt parameter, and *β_n_* = branching ratio).

BZOE-2	*A*_R_ (s^−1^)	*A*_NR_ (s^−1^)	*η*(%)	*Ω*_2_ (×10^−20^)	*Ω*_4_ (×10^−20^)	*β*_1_(%)	*β*_2_(%)	*β*_4_(%)	*Ω*_2_/*Ω*_4_
**λ_ex_ = 279 nm**	212.77	787.4	21.3	1.04	0.917	23.5	42.4	18.6	1.13
**λ_ex_ = 395 nm**	369	2331	13.7	2.27	2.78	13.6	53.7	32.5	0.82

## Data Availability

Data is contained within the article or supplementary materials.

## Supplementary Materials

The following are available online at https://www.mdpi.com/article/10.3390/nano12173028/s1, Experimental details. Figure S1. Size distribution plot for BZOE for different europium concentration derived using ImageJ software; Figure S2: Rietveld refinements for the BZOE samples; Figure S3: Ba L_3_ edge fits for the three Eu-doped samples; Table S1: Fit parameters for Ba L_3_ edge fits; Figure S4: Zr K edge fits for the three Eu-doped samples; Table S2: Fit parameters for Zr K edge fits; Figure S5: Eu L_3_ edge fits for the three Eu-doped samples; Figure S6: PL decay profiles of the various BZOE sample under excitation wavelengths of 279 nm and emission wavelengths of 625 nm corresponding ^5^D_0_→^7^F_2_ transitions of Eu^3+^ ions. References [34,54,55,56,57] are cited in the Supplementary Materials.
