# Supplementary material for: Excitation-Dependent Photoluminescence of BaZrO3:Eu3+ Crystals
Source: Nanomaterials (Basel). 2022 Aug 31;12(17):3028. doi: 10.3390/nano12173028 (PMC9457899; doi:10.3390/nano12173028)
Supplement: Supplementary file 1 [file nanomaterials-12-03028-s001.zip › nanomaterials-1855736-supplementary.pdf]

Electronic Supplementary Information

Excitation-Dependent Photoluminescence of $BaZrO_3:Eu^{3+}$ Crystals

Santosh K. Gupta[1,2], Hisham Abdou[3], Carlo U. Segre[4], and Yuanbing Mao[5,*]

[1]Radiochemistry Division, Bhabha Atomic Research Centre, Trombay, Mumbai-400085, India

[2]Homi Bhabha National Institute, Anushakti Nagar, Mumbai-400094, India

[3]Department of Chemistry, University of Texas Rio Grande Valley, 1201 West University Drive, Edinburg, Texas 78539, USA

[4]Center for Synchrotron Radiation Research and Instrumentation and Department of Physics, Illinois Institute of Technology, Chicago, IL 60616, USA

[5]Department of Chemistry, Illinois Institute of Technology, 3105 South Dearborn Street, Chicago, IL 60616, USA

*To whom correspondence should be addressed. Email: ymao17@iit.edu, Tel.: +1-312-567-3815

S1. Synthesis

Six $Ba_{1-x}ZrO_3$:x%Eu^{3+} (x = 0, 0.5, 1, 2, 5, 10) samples were synthesized using a molten salt synthesis method following a previously published procedure.[1] Specifically, commercially available barium oxalate (BaC_2O_4, 99.999%), europium nitrate pentahydrate ($Eu(NO_3)_3 \cdot 5H_2O$, 99.9%),zirconium(IV) oxide (ZrO_2, 99.978%), sodium hydroxide pellets (NaOH, 97.0%), and potassium hydroxide flakes (KOH, 90%) were used as received with no further purification. The starting reagents of BaC_2O_4, $Eu(NO_3)_3 \cdot 5H_2O$, ZrO_2, NaOH, and KOH were mixed in a stoichiometric ratio of 1-x : x : 1 : 9.8 : 10.2 and finely ground using a mortar and pestle inside a glove box. The resultant mixture was then transferred to an alumina crucible and isothermally heated in a muffle furnace at 820°C for 6 hours with ramp-up and ramp-down rates of 5°C/min. The formed product was then purified by filtration and washing with distilled water (18.2 MΩ at 25°C) several times, and then dried in an oven at 90°C overnight. The synthesized $Ba_{1-x}ZrO_3$:x%Eu^{3+} samples with x = 0, 0.5, 1, 2, 5, 10are designated as BZO, BZOE-0.5, BZOE-1, BZOE-2, BZOE-5 and BZOE-10, respectively.

S2. Characterization

The purity, morphology and structural properties of the obtained BZOE submicron crystals were characterized by powder X-ray diffraction (XRD), Raman spectroscopy, Fourier-transform infrared spectroscopy (FTIR), and scanning electron microscopy (SEM). A Rigaku MiniFlex X-ray diffractometer with a Cu $K_{\alpha 1}$ radiation (λ = 0.15406 nm, 30kV and 15mA) was used to obtain XRD patterns where 2-theta was used as scanning mode with a scanning range between 20° and 90°. Lattice parameters and isotropic crystallite sizes were obtained from the XRD patterns using GSAS-II.[2] A Bruker SENTERRA Raman spectrometer (Bruker Optics SENTERRA R200) that is equipped with an argon laser (785 nm) at 100mW power was used to record the Raman spectra of the samples. The FTIR spectra were recorded using a FT-IR ALPHA II's Platinum ATR single reflection diamond ATR module instrument. The scanning step size was set at 0.05° with a scanning rate of 2°/min. SEM images were collected using a Carl Zeiss sigma VP scanning electron microscope with a field emission gun that was set at 5kV.

The optical properties of the BZOE submicron crystals were studied using time resolved photoluminescence spectroscopy (TRPS), quantum yield measurements, and X-ray excited optical luminescence (XEOL). An Edinburgh Instrument FLS 980 fluorimeter system, that is equipped with a steady state and a pulsed xenon lamp sources with a pulse frequency range of 1-100 Hz, a 150 mm BenFlect coated integrating sphere, and a silver X-ray source tube with a power of 12 W (60 kV and 200 µA), was used to record the excitation and emission photoluminescence spectra, life time spectra, quantum yield, and radioluminescence spectra.

Synchrotron-radiation-based X-ray absorption near-edge structure (XANES) and EXAFS studies were carried out on the BZO and BZOE samples with doping concentrations of 1, 2 and 10% at the Zr K-edge and Ba and Eu L_3-edges.The XANES and EXAFS measurements were carried out at the Materials Research Collaborative Access Team (MRCAT) Sector 10-BM beamline at the Advanced Photon Source of Argonne National Laboratory.[3] Pellets of each sample were prepared with by mixing an optimal amount of sample with boron nitride and PVDF binder. EXAFS measurements were performed in transmission for the Zr K-edge (17998 eV) and fluorescence for both the Ba L_3-edge (5247 eV) and the Eu L_3-edge (6977 eV).

The X-ray absorption data were merged, normalized, background subtracted, Fourier Transformed, and fitted with structural models using the Athena and Artemis software which are part of the IFEFFIT package.[4,5]

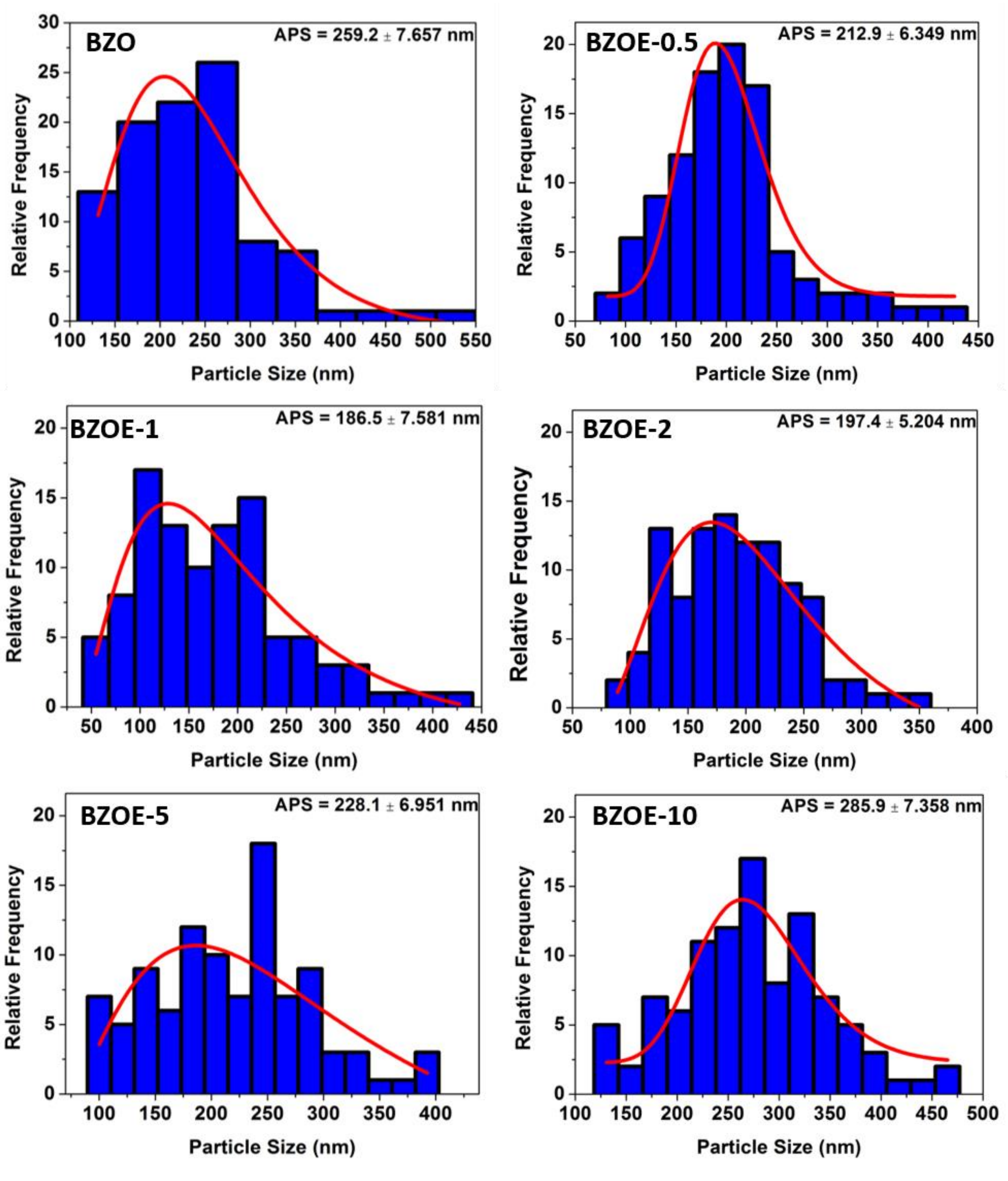

Figure S1. Size distribution plot for BZOE for different europium concentration derived using ImageJ software.

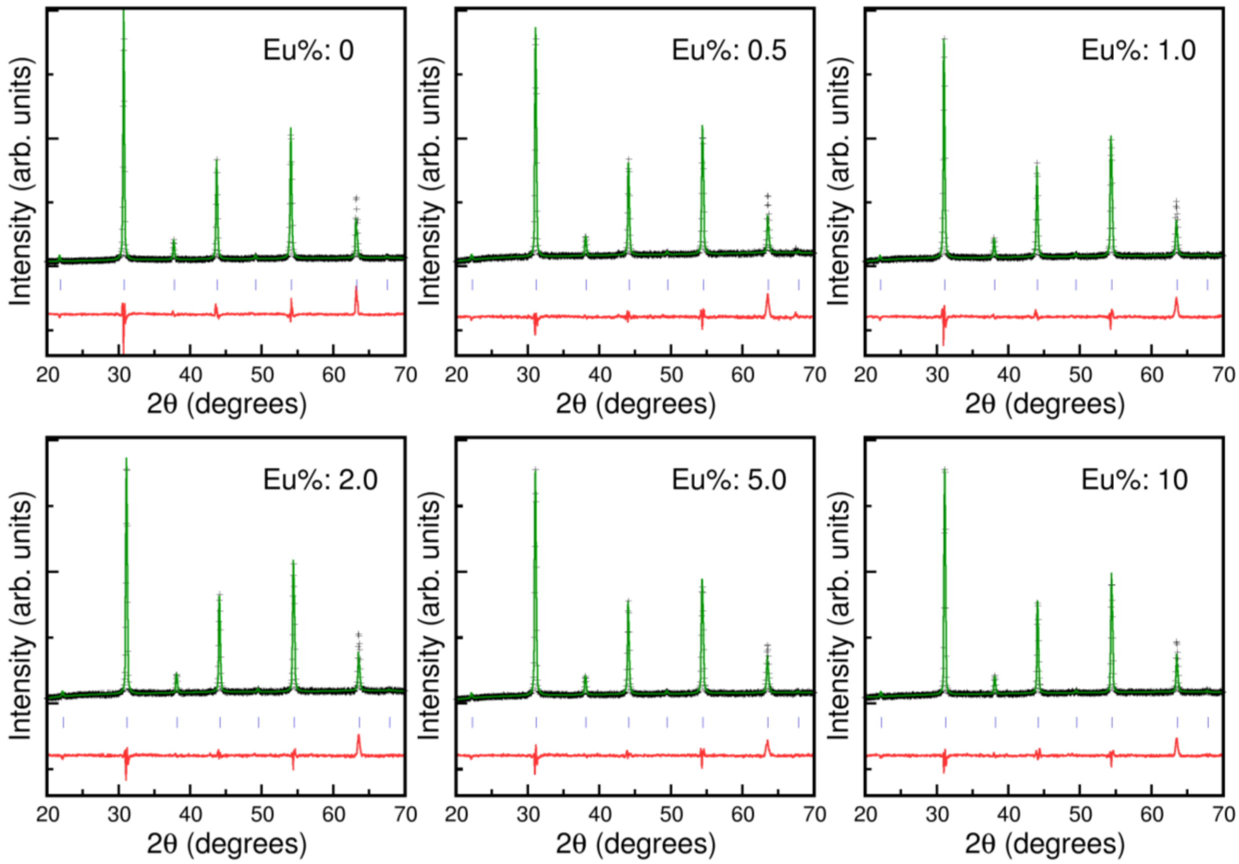

Figure S2. Rietveld refinements for the BZOE samples.

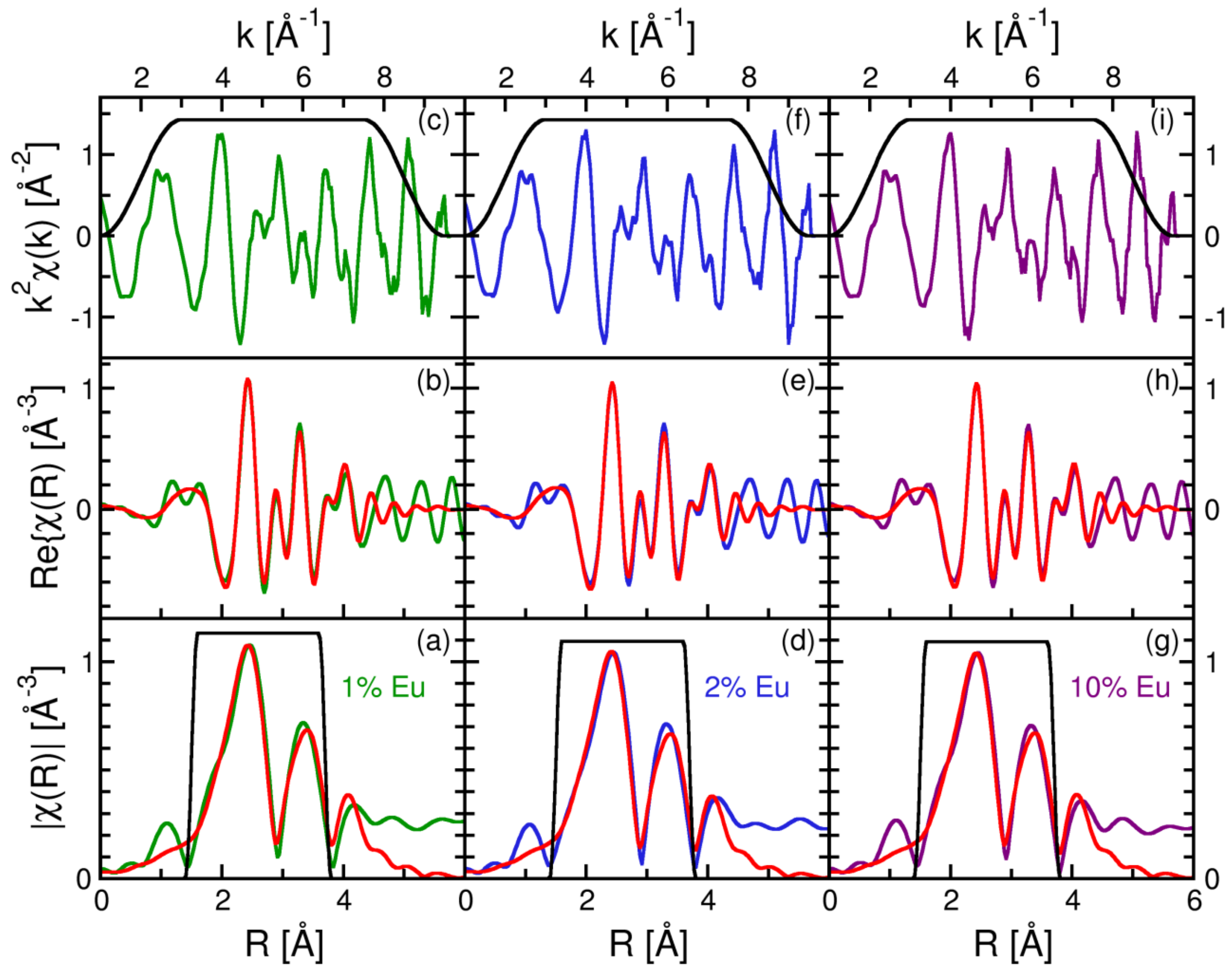

Figure S3. Ba L_3 edge fits for the three Eu-doped samples.

Table S1. Fit parameters for Ba L_3 edge fits

Scattering path	parameter	BZO	BZOE-1	BZOE-2	BZOE-10
Ba-O N=12	S_0^2	0.74±0.16	0.78±0.18	0.80±0.17	0.78±0.17
	R (Å)	2.91±0.02	2.91±0.02	2.91±0.02	2.91±0.02
	σ^2	0.011±0.005	0.013±0.005	0.013±0.005	0.013±0.005
Ba-Zr N=8	R (Å)	3.65±0.01	3.65±0.02	3.65±0.01	3.65±0.01
	σ^2	0.005±0.002	0.005±0.003	0.005±0.002	0.005±0.002
Ba-Ba N=6	R (Å)	4.23±0.08	4.22±0.08	4.22±0.07	4.22±0.07
	σ^2	Constrained to be same as for Ba-Zr path			

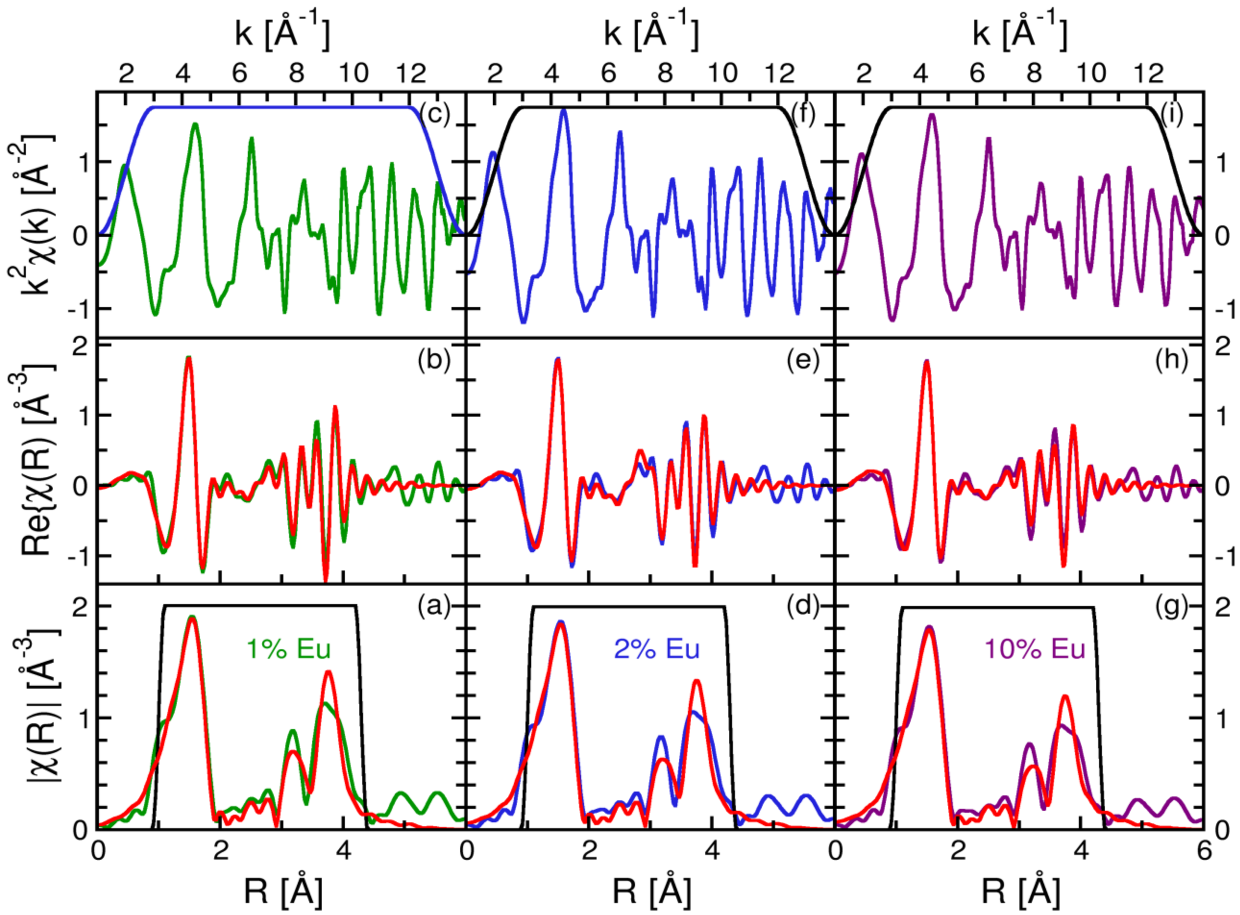

Figure S4. Zr K edge fits for the three Eu-doped samples.

Table S2. Fit parameters for Zr K edge fits

Scattering path	parameter	BZO	BZOE-1	BZOE-2	BZOE-10
Zr-O N=6	S_0^2	0.9±0.1	1.0±0.1	1.0±0.1	1.1±0.1
	R (Å)	2.10±0.01	2.10±0.01	2.10±0.01	2.11±0.01
	σ^2	0.004±0.001	0.005±0.002	0.005±0.002	0.006±0.002
Zr-Ba N=6	*R* (Å)	3.64±0.03	3.64±0.01	3.65±0.01	3.65±0.01
	σ^2	0.011±0.003	0.007±0.001	0.008±0.001	0.009±0.001
Zr-Zr N=8	*R* (Å)	4.21±0.02	4.22±0.02	4.23±0.02	4.23±0.02
	σ^2	0.002±0.002	0.002±0.001	0.002±0.001	0.002±0.001
Multiple Scatt. N=12,6,6	*R* (Å)	Constrained to be same as for Zr-Zr path			
	σ^2	0.006±0.001	0.006±0.001	0.007±0.001	0.008±0.002

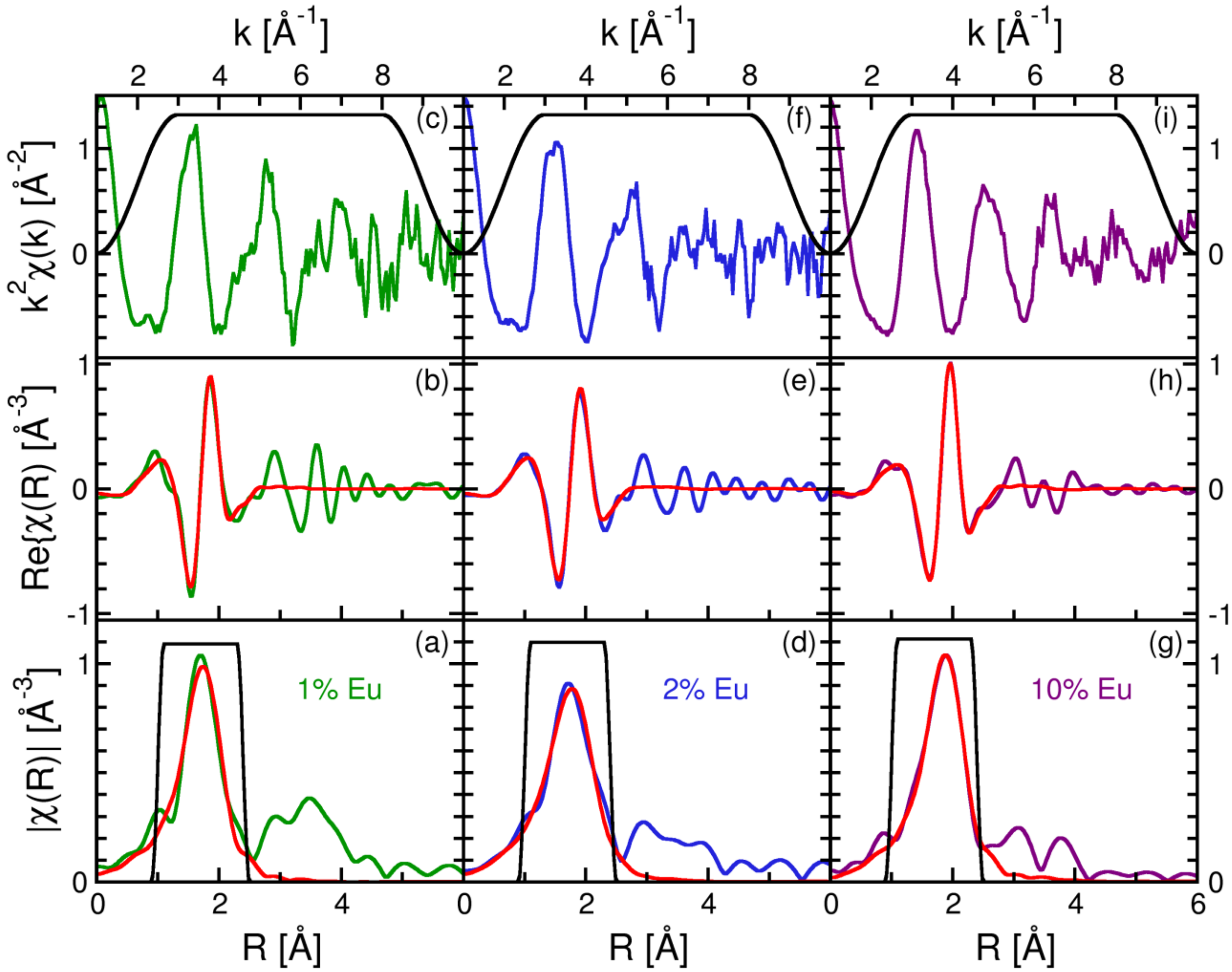

Figure S5. Eu L_3 edge fits for the three Eu-doped samples.

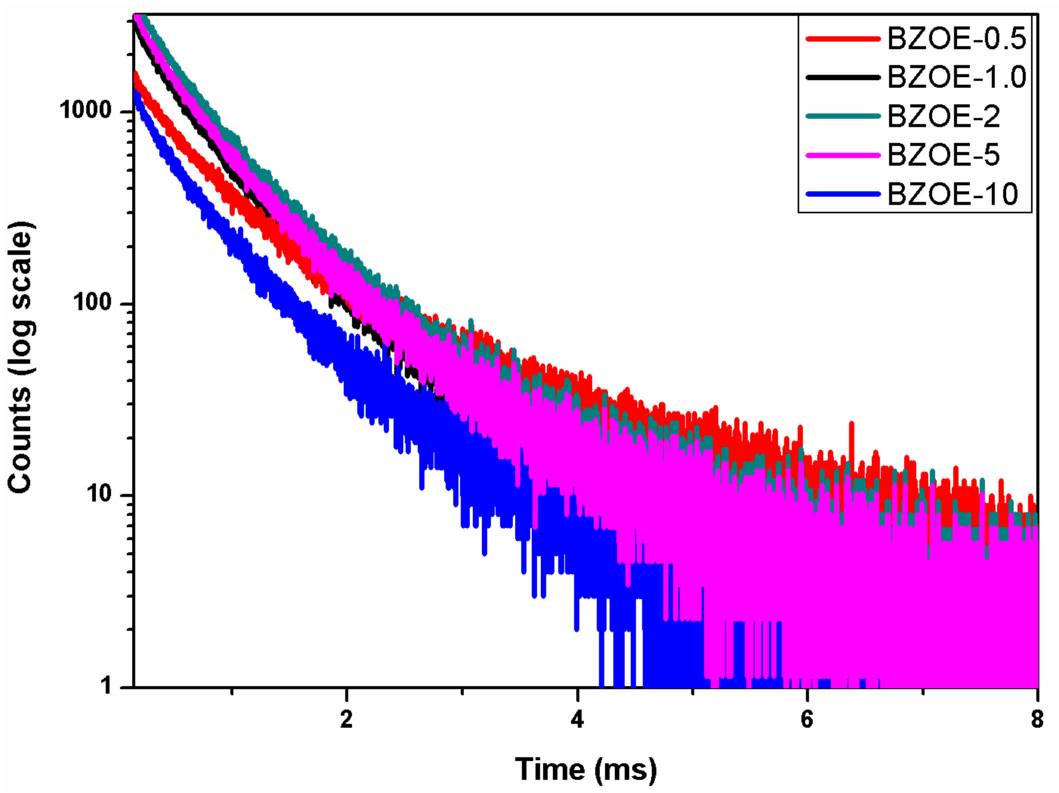

Figure S6. PL decay profiles of the various BZOE sample under excitation wavelengths of 279 nm and emission wavelengths of 625 nm corresponding $^5D_0 \rightarrow ^7F_2$ transitions of Eu^{3+} ions.

References:

[1] H. Zhou, Y. Mao, S.S. Wong, Shape control and spectroscopy of crystalline BaZrO3 perovskite particles, Journal of Materials Chemistry 17 (2007) 1707-1713.

[2] B. H. Toby, R. B. Von Dreele, GSAS-II: the genesis of a modern open-source all purpose crystallography software package, Journal of Applied Crystallography, 46(2), (2013) 544-549.

[3] A. Kropf, J. Katsoudas, S. Chattopadhyay, T. Shibata, E. Lang, V. Zyryanov, B. Ravel, K. McIvor, K. Kemner, K. Scheckel, The new MRCAT (Sector 10) bending magnet beamline at the advanced photon source, AIP Conference Proceedings, AIP, 2010, pp. 299-302.

[4] M. Newville, IFEFFIT: interactive XAFS analysis and FEFF fitting, Journal of Synchrotron Radiation 8 (2001) 322-324.

[5] B. Ravel, M. Newville, ATHENA, ARTEMIS, HEPHAESTUS: data analysis for X-ray absorption spectroscopy using IFEFFIT, Journal of Synchrotron Radiation 12 (2005) 537-541.
